# Enhanced Enterovirus D68 Replication in Neuroblastoma Cells Is Associated with a Cell Culture-Adaptive Amino Acid Substitution in VP1

**DOI:** 10.1128/mSphere.00941-20

**Published:** 2020-11-04

**Authors:** Syriam Sooksawasdi Na Ayudhya, Adam Meijer, Lisa Bauer, Bas Oude Munnink, Carmen Embregts, Lonneke Leijten, Jurre Y. Siegers, Brigitta M. Laksono, Frank van Kuppeveld, Thijs Kuiken, Corine GeurtsvanKessel, Debby van Riel

**Affiliations:** a Department of Viroscience, Erasmus MC, Rotterdam, The Netherlands; b National Institute for Public Health and the Environment, Centre for Infectious Diseases Research, Diagnostics and Laboratory Surveillance, Bilthoven, The Netherlands; c Virology Division, Department of Infectious Diseases and Immunology, Faculty of Veterinary Medicine, Utrecht University, Utrecht, The Netherlands; University of Pittsburgh School of Medicine

**Keywords:** VP1, cell culture adaptation, enterovirus D68, heparan sulfate, *in vitro*, neuroblastoma cells, neurotropism, pathogenesis, replication

## Abstract

Since its emergence in the United States in 2014, enterovirus D68 (EV-D68) has been and is associated with severe respiratory diseases and acute flaccid myelitis. Even though EV-D68 has been shown to replicate in different neuronal cells *in vitro*, it is currently poorly understood which viral factors contribute to the ability to replicate efficiently in cells of the central nervous system and whether this feature is a clade-specific feature. Here, we determined the replication kinetics of clinical EV-D68 isolates from (sub)clades A, B1, B2, B3, and D1 in human neuroblastoma cells (SK-N-SH). Subsequently, we compared sequences to identify viral factors associated with increased viral replication. All clinical isolates replicated in SK-N-SH cells, although there was a large difference in efficiency. Efficient replication of clinical isolates was associated with an amino acid substitution at position 271 of VP1 (E271K), which was acquired during virus propagation *in vitro*. Recognition of heparan sulfate in addition to sialic acids was associated with increased attachment, infection, and replication. Removal of heparan sulfate resulted in a decrease in attachment, internalization, and replication of viruses with E271K. Taken together, our study suggests that the replication kinetics of EV-D68 isolates in SK-N-SH cells is not a clade-specific feature. However, recognition of heparan sulfate as an additional receptor had a large effect on phenotypic characteristics *in vitro.* These observations emphasize the need to compare sequences from virus stocks with clinical isolates in order to retrieve phenotypic characteristics from original virus isolates.

**IMPORTANCE** Enterovirus D68 (EV-D68) causes mild to severe respiratory disease and is associated with acute flaccid myelitis since 2014. Currently, the understanding of the ability of EV-D68 to replicate in the central nervous system (CNS), and whether it is associated with a specific clade of EV-D68 viruses or specific viral factors, is lacking. Comparing different EV-D68 clades did not reveal clade-specific phenotypic characteristics. However, we did show that viruses which acquired a cell culture-adapted amino acid substitution in VP1 (E271K) recognized heparan sulfate as an additional receptor. Recognition of heparan sulfate resulted in an increase in attachment, infection, and replication in neuroblastoma cells compared with viruses without this specific amino acid substitution. The ability of EV-D68 viruses to acquire cell culture-adaptive substitutions which have a large effect in experimental settings emphasizes the need to sequence virus stocks.

## Abstract (precis)

Enterovirus D68 (EV-D68) causes mild to severe respiratory disease and is associated with acute flaccid myelitis since 2014. Currently, the understanding of the ability of EV-D68 to replicate in the central nervous system (CNS), and whether it is associated with a specific clade of EV-D68 viruses or specific viral factors, is lacking. Comparing different EV-D68 clades did not reveal clade-specific phenotypic characteristics. However, we did show that viruses which acquired a cell culture-adapted amino acid substitution in VP1 (E271K) recognized heparan sulfate as an additional receptor. Recognition of heparan sulfate resulted in an increase in attachment, infection, and replication in neuroblastoma cells compared with viruses without this specific amino acid substitution. The ability of EV-D68 viruses to acquire cell culture-adaptive substitutions which have a large effect in experimental settings emphasizes the need to sequence virus stocks.

## INTRODUCTION

Enterovirus D68 (EV-D68) is known to cause mild to severe respiratory infections, but since 2014 it has been increasingly associated with neurological complications (1–3). After its discovery in 1962, cases were only sporadically reported (4). EV-D68 caused a large outbreak of severe respiratory disease across the United States of which about 10.4% of symptomatic cases developed acute flaccid myelitis (AFM) from August 2014 to January 2015 (5). During this outbreak, more than 2,000 cases of EV-D68 infections were reported in 20 countries (6–9), of which about 7% of symptomatic cases developed AFM (10). Other neurological complications like cranial nerve dysfunction and encephalitis were occasionally reported (11–13). Since then, biennial epidemics of EV-D68 occur globally, with the largest epidemic in 2018 (14–17).

EV-D68 belongs to the *Enterovirus* genus in the *Picornaviridae* family. It is a nonenveloped, positive-sense, single-stranded RNA virus. Within the capsid, the structural protein VP1 plays an important role in the attachment to host cells, and its gene is the most variable part of the genome and used for genotyping (18). Circulating EV-D68 isolates use cell surface glycoproteins including sialylated glycoproteins or glycolipid as a receptor. These sialylated receptors bind to the canyon in VP1, which leads to conformational change and subsequent dysregulation of stability and thereby initiates uncoating (19). However, recent studies have identified nonsialylated receptors, such as ICAM-5 and heparan sulfate glycosaminoglycans (GAGs), for specific EV-D68 isolates (20, 21).

To date, EV-D68 is divided into four clades, from A to D, although the initial division in three clades, A, B, and C, with subclades A1 and A2, is also used. Some researchers use A for A1 and D for A2 (22, 23). EV-D68 clade B is subdivided into subclades B1, B2, and B3 (24), and clade D has recently been subdivided into subclades D1 and D2 (25). Multiple clades circulated during the 2014 outbreak, but subclade B1 was the most prevalent and was also associated with neurological complications. Based on this observation, it was initially thought that the ability to invade and replicate in the central nervous system (CNS) was a recently acquired feature and clade specific (2, 3, 8, 10, 26). However, since 2016, subclade B3 and to a lesser extent subclade D1 became predominant, and both were associated with neurological complications (16, 27–29). In addition, recent studies have shown that multiple isolates from different clades are able to infect both neuronal cells and neuroblastoma cell lines (30) and that non-subclade B1 and B3 isolates were able to cause paralysis in mice after intracranial inoculation. Interestingly, isolates from the same clade differed in their ability to cause paralysis in mice (31). Altogether, this suggests that the ability to invade and replicate in the CNS is not a clade-specific feature. Even though several studies have determined the *in vitro* phenotypic characteristics of EV-D68 isolates in cells of the CNS, viral factors associated with efficient attachment, infection, and replication have not been identified yet. In addition, genetic analysis of EV-D68 isolates associated with phenotypic characteristics *in vitro* has not been carried out in previous studies.

In this study, we investigated the ability of EV-D68 isolates from different clades to attach and infect human neuroblastoma cells (SK-N-SH) and determined their replication kinetics. Subsequently, we compared viral sequences in order to identify viral factors associated with increased viral replication.

## RESULTS

### Clinical enterovirus D68 isolates from different clades replicate in human neuroblastoma cells with different efficiency.

**FIG 1:**
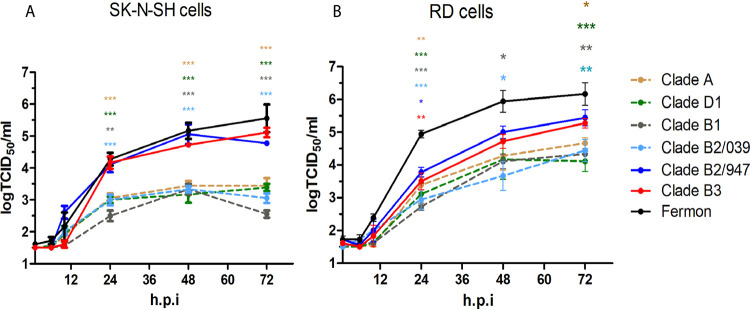
Growth curves of EV-D68 isolates in SK-N-SH and RD cells. Growth curve of clinical isolates of (sub)clades A, B1, B2, B3, and D1 and prototype EV-D68 strain Fermon in SK-N-SH cells (A) and RD cells (B) at an MOI of 0.01. Statistical analysis was performed using the one-way analysis of variance (ANOVA) compared to Fermon. Data are shown as mean ± SD from three independent experiments. *, *P* ≤ 0.05; **, *P* ≤ 0.01; ***, *P* ≤ 0.001.

Growth curves using a multiplicity of infection (MOI) of 0.01 were generated for clinical isolates of clade A (or A1; here we use A), subclade B1, subclade B2 (B2/039 and B2/947), subclade B3, subclade D1 (or A2; here we use D1), and the prototype Fermon strain in SK-N-SH and rhabdomyosarcoma (RD) cells. Growth curves showed that all viruses replicated in SK-N-SH cells, but virus titers of subclades B2/947 and B3 and Fermon were significantly higher than those of clade A and subclades D1, B1, and B2/039 from 24 h postinoculation (hpi) onward (Fig. 1A). Remarkable, large differences between the two subclade B2 isolates were observed in growth kinetics on SK-N-SH cells. The same pattern was observed in RD cells, but the differences among isolates were smaller. Isolates that replicated most efficiently on SK-N-SH cells also did so in RD cells (Fig. 1B). Overall, these data show that EV-D68 isolates from all clades are able to replicate in SK-N-SH and RD cells but do so with different efficiency.

### Replication efficiency is associated with percentage of infection.

**FIG 2:**
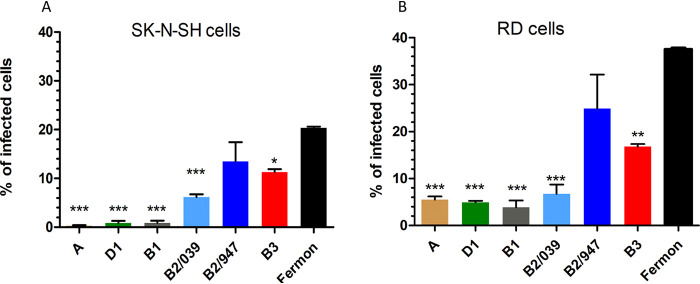
Infection efficiency of different EV-D68 isolates in SK-N-SH and RD cells. Percentage of infected SK-N-SH cells (A) and RD cells (B) after infection with clinical isolates from A, B1, B2, B3, and D1 and prototype EV-D68 strain Fermon 8 h postinfection (MOI of 2). The infected cells were stained for VP1 antigen using immunofluorescence staining. Three high-power fields were captured per sample. Statistical analysis was performed using the one-way ANOVA in comparison with Fermon. Data are shown as mean ± SD from three independent experiments. *, *P* ≤ 0.05; **, *P* ≤ 0.01; ***, *P* ≤ 0.001.

To study whether the replication efficiency was associated with the infection efficiency of the different EV-D68 isolates, the percentage of infection was determined by immunofluorescence using an anti-VP1 antibody after incubation of viruses on SK-N-SH and RD cells with an MOI of 2 for 8 h. Viruses of clade B2/947 and Fermon, and to a lesser extent B3, which replicated most efficiently in SK-N-SH and RD cells, infected higher percentages of cells than did other isolates (Fig. 2A and B). These results suggest that the efficient replication measured by the growth kinetics was associated with efficient infection of cells.

### Amino acid differences between viruses of subclade B2.

Since we observed a large difference in the replication kinetics between the two isolates of subclade B2, we initially performed full genome sequencing analysis of the stocks from B2/039 and B2/947 isolates. There were 13 amino acid differences between these two viruses: V66A, R67K, K116R, V166I, R234G, V136M, D140N, E271K, R25K, I37V, K56R, I187V, and S56R (Table 1). These amino acid differences were present in both structural proteins (VP4, VP2, VP3, and VP1) and nonstructural proteins (2A, 3C, and 3D).

**TABLE 1:** Amino acid differences between clade B2/947 and clade B2/039 isolates

Isolate	Amino acid at position in protein												
	VP4	VP2		VP3		VP1			2A		3C		3D
	66	67	116	166	234	136	140	271	25	37	56	187	56
B2/947	A	K	R	I	G	M	N	K	K	V	R	V	R
B2/039	V	R	K	V	R	V	D	E	R	I	K	I	S

### A lysine is present at position 271 of capsid protein VP1 in clinical isolates that replicate efficiently in SK-N-SH and RD cells.

After the identification of amino acid differences between the two clinical isolates of subclade B2, the 13 amino acid positions that differed were verified in the other clinical isolates to identify amino acid substitutions possibly associated with increased infection and replication efficiency. A lysine (K) at position 271 in the VP1 capsid protein was observed only in clade B2/947 and B3 viruses, the two clinical isolates that replicated most efficiently in SK-N-SH and RD cells. All other clinical isolates had a glutamic acid (E) or valine (V) at position 271 in VP1, and the lab-adapted Fermon carried an aspartic acid (D) (Table 2). At all other positions that differed between the clade B2 isolates, no common differences were observed between the viruses with different replication efficiencies (Table 2). This indicates that the amino acid substitution E271K might be a determinant for increased infection and replication in SK-N-SH cells *in vitro.*

**TABLE 2:** Amino acids present in clinical isolates of (sub)clades A, B1, B2, B3, and D1 and Fermon at the positions that differed between low- and high-replicating isolates

Isolate	Amino acid at position in protein												
	VP4	VP2		VP3		VP1			2A		3C		3D
	66	67	116	166	234	136	140	271	25	37	56	187	56
B2/947^*a*^	A	K	R	I	G	M	N	K	K	V	R	V	R
B3^*a*^	A	R	K	I	G	V	D	K	R	I	R	V	S
B2/039	V	R	K	V	R	V	D	E	R	I	K	I	S
A	A	K	K	V	R	T	D	E	R	V	K	V	S
D1	A	K	K	V	A	M	D	E	R	I	K	V	S
B1	A	K	K	I	G	V	D	V	R	V	R	V	S
Fermon^*a*^	A	R	K	I	G	M	D	D	R	V	R	V	S

a Isolates that replicate efficiently in SK-N-SH cells.

### A lysine at position 271 in VP1 is acquired during *in vitro* passaging.

The clinical isolates used in this study were passaged in RD cells to produce virus stocks. To investigate whether a lysine at position 271 in VP1 was present in the original isolate of the patient, sequences from clinical specimens, previous passages, and virus stocks used in this study were generated and aligned. In the clinical specimens, a glutamic acid (E) was found at position 271 in VP1 of both subclade B2/947 (21) and subclade B3. Furthermore, sequencing analysis of all virus passages showed the acquisition of a lysine (K) in passage 3 of subclade B2/947 and passage 2 of subclade B3 on RD cells (Table 3). There were no minor variants present at position 271 of sequences derived from clinical specimens and stock B2/947 and B3 viruses using a 20% cutoff (Table 3). This indicates that viruses with high replication and infection efficiency acquired the E271K amino acid substitution during passaging in RD cells.

**TABLE 3:** Amino acid at position 271 of VP1 in isolates B2/947 and B3 from original clinical specimens and historical passages

Isolate	Clinical specimen/virus isolate passage	Amino acid at position 271	Frequent variant; method	GenBank accession no.
B2/947	Clinical specimen	E	100%; Illumina	
	Passage 1	Position not sequenced		
	Passage 2	E	100%; Sanger	KT231897
	Passage 3	K	100%; Sanger	KT231898
	Passage 4	K	100%; Illumina	MN954540

B3	Clinical specimen	E	100%; Sanger	
	Passage 1	E	100%; Sanger	
	Passage 2	K	100%; Illumina	
	Passage 3	K	100%; Illumina	
	Passage 4	K	100%; Illumina	MN954541

### Viruses with the E271K substitution in VP1 attached more efficiently to SK-N-SH and RD cells.

**FIG 3:**
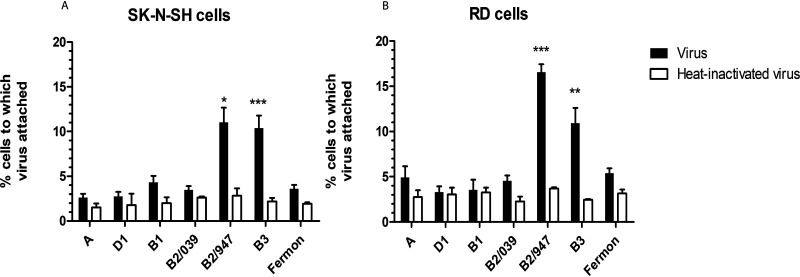
Virus attachment of EV-D68 viruses to SK-N-SH and RD cells. Experiments were performed using flow cytometry. Attachment of clinical isolates from (sub)clades A, B1, B2, B3, and D1 and prototype strain Fermon to SK-N-SH cells (A) and RD cells (B). Heat-inactivated viruses were included as negative control. Statistical analysis was performed using the one-way ANOVA in comparison to Fermon. Data are shown as mean ± SD from three independent experiments. *, *P* ≤ 0.05; **, *P* ≤ 0.01; ***, *P* ≤ 0.001.

Since the amino acid at position 271 of EV-D68 resides within the canyon, close to the sialic acid binding site (19), we investigated whether EV-D68 isolates with the specific E271K amino acid substitution attached to higher percentages of SK-N-SH and RD cells. We observed that the subclade B2/947 and subclade B3 isolates which acquired the E271K substitution attached to higher percentages of SK-N-SH and RD cells than did all other isolates. Fermon attached to lower percentages of SK-N-SH and RD cells despite efficient replication and infection (Fig. 3A and B). Overall, these observations suggest that the E271K substitution is associated with increased virus attachment *in vitro*.

### Viruses with E271K substitution in VP1 recognize sialic acid and heparan sulfate.

**FIG 4:**
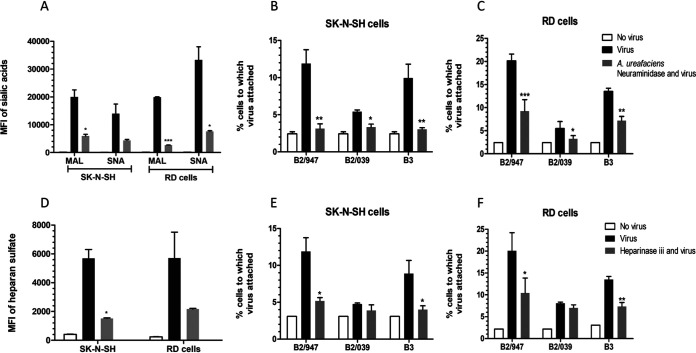
Virus attachment of clinical isolates B2/039, B2/947, and B3 after neuraminidase and heparinase III treatment. (A) Mean fluorescent intensity (MFI) levels of MAL [α(2,3)-linked sialic acid] and SNA [α(2,6)-linked sialic acid] with and without neuraminidase treatment. (B and C) Percentage of attachment of isolates B2/039, B2/947, and B3 with and without neuraminidase treatment of SK-N-SH cells (B) and RD cells (C). (D) MFI levels of heparan sulfate with and without heparinase III treatment. (E and F) Attachment of clinical isolates B2/039, B2/947, and B3 with and without heparinase III treatment on SK-N-SH cells (E) and RD cells (F). Statistical analysis was performed by paired *t* test. Data are shown as mean ± SD from three independent experiments. *, *P* ≤ 0.05; **, *P* ≤ 0.01; ***, *P* ≤ 0.001.

A previous study has shown that among several EV-D68 strains, subclade B2/947 can recognize heparan sulfate as an additional receptor to sialic acid (21). To investigate whether our viruses with an E271K substitution were able to recognize both sialic acid and heparan sulfate, we determined the ability of these viruses to attach to cells that were pretreated with Arthrobacter ureafaciens neuraminidase and heparinase III to remove sialic acids or heparan sulfate, respectively. Subclade B2/947 and B3 isolates carrying the E271K substitution, and the subclade B2/039 isolate (271E) as control, were included in these analyses. Removal of sialic acids and heparan sulfate was confirmed by measuring mean fluorescent intensity (MFI) with Maackia amurensis lectin (MAL) for α(2,3)-linked sialic acid and Sambucus nigra lectin (SNA) α(2,6)-linked sialic acid, and with an antibody that recognizes heparan sulfate (Fig. 4A and D). The percentage of cells to which the virus attached was significantly decreased for all viruses in both SK-N-SH and RD cells after neuraminidase treatment (Fig. 4B and C). In contrast, heparinase III treatment resulted in decreased attachment of only viruses with the E271K substitution (Fig. 4E and F). Overall, these results showed that the viruses with the E271K substitution recognize both sialic acid and heparan sulfate on the cellular surface.

### Attachment, internalization, and replication of viruses with E271K substitution in VP1 depend in part on heparan sulfate recognition.

**FIG 5:**
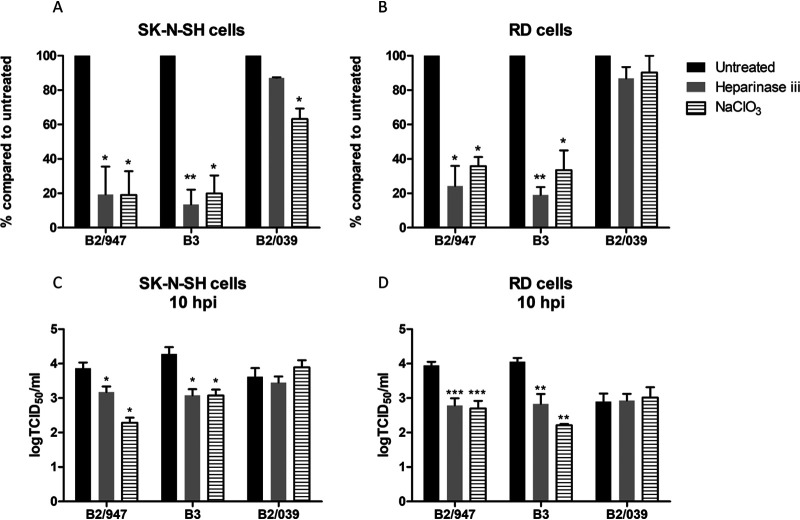
Attachment, internalization, and replication of clinical isolates B2/947, B3, and B2/039 in SK-N-SH and RD cells after heparinase III and NaClO_3_ treatment. SK-N-SH and RD cells were treated for 1 h with heparinase III, or cells were cultured for 5 days with NaClO_3_. After the treatment, cells were infected with clinical isolates at an MOI of 1. To remove the unbound viruses, cells were washed 3 times with PBS before cell lysis. Attached and intracellular viral RNA levels were determined by qPCR 1 h postinfection. Percentages of viral RNA levels were compared to values for untreated cells in SK-N-SH cells (A) and RD cells (B). Infectious virus particles of the supernatants from SK-N-SH cells (C) and RD cells (D) were determined by TCID_50_ 10 h postinfection. Statistical analysis was performed using the one-way ANOVA. Data are shown as mean ± SD from three independent experiments. *, *P* ≤ 0.05; **, *P* ≤ 0.01; ***, *P* ≤ 0.001.

To investigate if heparan sulfate is required for attachment, internalization, and replication of viruses with an E271K substitution, heparan sulfate was removed on SK-N-SH and RD cells by either heparinase III or NaClO_3_, which prevents cell surface sulfation (21). Treated and untreated cells were inoculated with an MOI of 1 of B2/947, B3, and B2/039. After 1 hpi at 37°C, the amount of attached and internalized viral RNA of B2/947 and B3 was smaller in the heparinase III- or NaClO_3_-treated cells than in untreated cells by qPCR. Viral RNA for B2/039 was not reduced in the heparinase- or NaClO_3_-treated cells, with the exception of a minimal but significant reduction of viral RNA in SK-N-SH cells treated with NaClO_3_ but not in RD cells (Fig. 5A and B). Virus replication of both B2/947 and B3 was significantly decreased in SK-N-SH and RD cells treated with heparinase III or NaClO_3_, while replication levels of B2/039 were similar in treated and untreated cells (Fig. 5C and D; see also Fig. S1 in the supplemental material). Together, these results indicate that the recognition of heparan sulfate is important for facilitating virus attachment, internalization, and replication.

10.1128/mSphere.00941-20.1 FIG S1 Growth curves of subclade B2/947, B3, and B2/039 isolates in SK-N-SH and RD cells after heparinase III and NaClO_3_ treatment. Growth curves of clinical isolates of subclades B2/947, B3, and B2/039 in SK-N-SH cells (A to C) and RD cells (D to F) at an MOI of 1. Statistical analysis was performed using the one-way ANOVA compared to untreated cells. Data are shown as mean ± SD from three independent experiments. *, *P* ≤ 0.05; **, *P* ≤ 0.01; ***, *P* ≤ 0.001. Download FIG S1, DOCX file, 0.2 MB. Copyright © 2020 Sooksawasdi Na Ayudhya et al. 2020 Sooksawasdi Na Ayudhya et al. This content is distributed under the terms of the Creative Commons Attribution 4.0 International license.

## DISCUSSION

Here, we report that EV-D68 isolates from (sub)clades A, B1, B2, B3, and D1 replicated in human neuroblastoma SK-N-SH cells; however, large differences were observed in replication efficiency. Subsequent genotypic analysis revealed that viruses replicating most efficiently had a specific amino acid substitution in VP1 (E271K). This substitution was acquired during cell culture propagation and was associated with the recognition of heparan sulfate. Furthermore, attachment, internalization, and replication depended in part on heparan sulfate recognition for viruses with the E271K substitution in VP1. These findings suggest that efficient replication of EV-D68 isolates *in vitro* in human neuroblastoma cells is not a clade-specific feature but at least in part associated with the usage of heparan sulfate as an additional receptor.

The amino acid substitution at position 271 of VP1 (E271K) acquired during *in vitro* passaging in 2 out of the 6 viruses included in our study was associated with the recognition of heparan sulfate as an additional receptor (21), which had a large impact on the phenotypic characteristics of EV-D68 viruses. Heparan sulfate is abundantly expressed on RD cells (32) and associated with increased virus attachment, infection efficiency, and virus replication in cells *in vitro*. The ability of EV-D68 to replicate in mouse neuroblastoma cells and human induced pluripotent stem cell motor neurons were independent of sialic acid recognition, although the usage of heparan sulfate was not investigated (30, 33). In our study, unfortunately, viruses from earlier passages without E271K in VP1 were not available and an infectious clone of the clinical isolate B2/947 with the original VP1 amino acid E271 could not be recovered (21), which made a direct comparison of viruses with and without E271K in VP1 not possible. The lysine at position 271 of VP1 is positively charged, which contributes to the formation of a basic patch that interacts with negatively charged heparan sulfate proteoglycan (21). This mechanism has been described previously for other picornaviruses which acquired the recognition of heparan sulfate during passaging of viruses *in vitro* (34–36).

It was initially thought that the neurotropic potential of EV-D68 was a feature recently acquired and was associated with subclade B1. However, epidemiological and clinical data have shown that different subclades, namely, B3 and D1, besides subclade B1 have also been associated with AFM cases after 2014 (16, 28, 29). In addition, most *in vitro* studies, including ours, and *in vivo* studies do not reveal phenotypic difference between clades or recent and older EV-D68 isolates (30, 31, 37). *In vivo*, EV-D68 isolates from multiple clades (e.g., clade A and subclade B1) were able to cause paralysis after intracranial inoculation (31). *In vitro*, we and others showed that EV-D68 isolates from multiple clades were able to replicate in neuroblastoma cells (37). Furthermore, we did not detect phenotypic differences between viruses isolated before and after 2014 independent of the clade, which correlates with the study by Rosenfeld et al. (30), but not with a study performed by Brown et al. (37). Unfortunately, virus stocks were not compared with clinical isolates in these studies. All together, these data suggest that the ability to replicate in cells of the CNS is not a clade-specific feature.

The role of heparan sulfate as an additional receptor in the pathogenesis *in vivo* remains unclear. Although the presence of the E271K substitution in human EV-D68 isolates has not been studied, we found that only a very few EV-D68 VP1 sequences available in GenBank have a lysine at position 271 of VP1 (by 15 December 2019, 17 out of 1,737 [<1%]), and they fall in a wide range of old and current clades. However, of most sequences the source, clinical material or virus isolate, and passage history are not known. Interestingly, acquisition of heparan sulfate recognition in an immunocompromised patient with enterovirus 71 (EV-71) was associated with systemic spread, including the CNS. In this patient a mutation in the VP1 responsible for heparan sulfate recognition was detected only in extrarespiratory samples (38). Since heparan sulfate is not expressed on the apical side of respiratory epithelial cells, but abundantly in cells outside the respiratory tract, such as cells of the CNS and muscle cells (38, 39), *in vivo* acquisition of E271K might—just like EV-71—occur outside the respiratory tract and would therefore hardly be found in respiratory samples. Sequence analysis of respiratory and extrarespiratory samples from the same patient should reveal if heparan sulfate recognition is important for the pathogenesis of EV-D68 *in vivo*.

It remains unclear why there has been an increase of AFM cases associated with EV-D68 since 2014, since epidemiological and serological studies suggest that EV-D68 has been circulating throughout the population for decades (40, 41). Currently, the pathogenesis of neurological disease associated with EV-D68 infection is not fully understood. Either a direct effect of virus entry and replication in the nervous system or an indirect effect of systemic cytokines could contribute. However, the detection of EV-D68 or virus-specific antibodies in the cerebrospinal fluid suggests that virus enters and replicates in the CNS in at least some of the patients (42, 43). As there have been more cases with severe respiratory disease caused by EV-D68 since 2014 (9, 44), it might be that recent EV-D68 viruses replicate to higher titers in the respiratory tract, thereby increasing the risk of systemic spread including that to the CNS. However, understanding the exact mechanism of systemic spread of EV-D68 *in vivo* requires more in-depth pathogenesis studies.

Taken together, we demonstrate that the replication of EV-D68 isolates in the neuroblastoma cell line SK-N-SH is not a clade-specific phenotype of EV-D68. However, large phenotypic differences that we did observe *in vitro* could be linked to the substitution E271K in VP1 leading to the recognition of heparan sulfate as an additional receptor, which resulted in increased attachment, internalization, and replication. Therefore, we recommend sequencing of virus stocks in order to obtain viruses that resemble clinical isolates in order to study phenotypic characteristics of EV-D68 isolates *in vitro* and *in vivo*.

## MATERIALS AND METHODS

### Cells.

Rhabdomyosarcoma (RD cells) (ATCC) were maintained in Dulbecco’s modified Eagle’s medium (DMEM; Lonza, Basel, Switzerland) supplemented with 10% (vol/vol) fetal calf serum (FCS; Sigma-Aldrich, St. Louis, MO, USA), 100 IU/ml penicillin (Lonza), 100 IU/ml streptomycin (Lonza), and 2 mM l-glutamine (Lonza), at 37°C with 5% CO_2_. SK-N-SH neuroblastoma cells (Sigma-Aldrich) were maintained in Eagle’s minimum essential medium (EMEM) and Earle’s balanced salt solution (EBSS) (Lonza), 10% (vol/vol) FCS, 100 IU/ml penicillin (Lonza), 100 μg/ml streptomycin (Lonza), 2 mM l-glutamine (Lonza), 1% nonessential amino acids (Lonza), 1 mM sodium pyruvate (Thermo Fisher Scientific, Waltham, MA, USA), and 1.5 mg/ml sodium bicarbonate (Lonza), at 37°C with 5% CO_2_. All SK-N-SH cells used in the study were from the same batch and maintained up to 15 passages.

### Viruses.

Enterovirus D68 viruses included in this study were isolated from clinical specimens at the National Institute of Public Health and the Environment (RIVM), Bilthoven, The Netherlands. The viruses were initially isolated on RD cells at 33°C at RIVM from patients with respiratory disease caused by EV-D68 infection. Virus stocks for the *in vitro* studies were grown at the Viroscience laboratory, Erasmus MC (EMC), Rotterdam, The Netherlands, in RD cells (ATCC) at 33°C in 5% CO_2._ The viruses included in this study, with virus reference number, year of collection and isolation, passage number, and accession number are as follows: clade A (or A1) (4311200821; 2012, passage RD3, accession number MN954536) (45), D subclade D1 (or A2) (4311400720; 2014, passage RD4, accession number MN954537) (45), B1 (4311300117; 2013, passage RD4, accession number MN954538 (45), B2/039 (4311201039; 2012, passage RD3, accession number MN954539) (45), B2/947 (4310900947; 2009, passage RD4, accession number MN954540 (19, 46), and B3 (4311601013; 2016, passage RD4, accession number MN954541). EV-D68 prototype Fermon was provided by Frank van Kupperveld, Utrecht University, Utrecht, The Netherlands.

### Virus titrations.

Virus titers (median tissue culture infectious dose [TCID_50_]) of the virus stocks were determined by endpoint titrations in RD cells. Briefly, 10-fold serial dilutions were made and inoculated onto a monolayer of RD cells. The inoculated plates were incubated at 33°C in 5% CO_2_. Cytopathic effect (CPE) was determined at day 5, and virus titers were determined using the Spearman-Kärber method (47).

### Replication kinetics, virus infection, and internalization.

Virus infection by EV-D68 isolates was determined using a multiplicity of infection (MOI) of 0.01 or 1. Monolayers of SK-N-SH and RD cells in a 6-well plate or a 96-well plate were inoculated with the different EV-D68 viruses for 1 h at 37°C in 5% CO_2_ or cell culture medium as a control. After 1 h of virus adsorption, the inoculum was removed, and cells were washed once with PBS before the cells were lysed with lysis buffer (Roche, The Netherlands) for qPCR analysis or replenished with culture medium and incubated at 37°C in 5% CO_2_. The supernatant was collected at the indicated hour postinoculation (hpi) and stored at −80°C for subsequent virus titration.

### Percentage of infections.

SK-N-SH and RD cells (∼80% confluent) in a 96-well plate were infected with EV-D68 at an MOI of 2. Mock-infected cells were included as a negative control. EV-D68-infected SK-N-SH and RD cells were incubated at 37°C and 33°C in 5% CO_2_, respectively. After 8 hpi, cells were fixed with 4% paraformaldehyde (PFA) for 20 min at room temperature, washed with PBS, and permeabilized with 70% ethanol. Cells were first incubated with 5% bovine serum albumin (BSA; Aurion, Wageningen, The Netherlands) in PBS for 30 min before incubation with rabbit anti-EV-D68 VP1 (20 μg/ml; GeneTex, Irvine, CA, USA) for 1 h. Cells were washed twice with PBS and incubated with goat anti-rabbit IgG conjugated with Alexa Fluor 594 (10 μg/ml; Life Technologies, Inc., The Netherlands) in PBS with 0.1% BSA (Aurion) for 1 h. Cells were washed 3 times with PBS and mounted with ProLong Diamond Antifade with DAPI (4′,6-diamidino-2-phenylindole; Life Technologies) to visualize nuclei. Each experiment included negative and omission controls. EV-D68 VP1-positive cells were identified using a Zeiss LSM 700 laser scanning microscope. All images were processed using Zen 2010 software. Per sample, 3 high-power fields were photographed, and the number of infected cells was calculated by counting virus-infected/uninfected cells in 3 randomly chosen panels. All experiments were performed in triplicate.

### Next-generation sequencing.

After pretreatment of the virus stock with OmniCleave (Lucigen, Halle-Zoersel, Belgium), RNA was extracted using the NucleoSpin RNA II kit (Bioke, Leiden, The Netherlands) according to the manufacturer’s instructions. First-strand cDNA was synthesized from using random hexamers and Superscript IV (Thermo Fisher Scientific). Double-stranded DNA was generated using Klenow fragment (New England Biolabs [NEB]). For library preparation, the Kapa HyperPlus library preparation kit (Roche, Basel, Switzerland) was used according to the manufacturer’s instructions with minor modifications. Adapters were diluted 1:10, and a second wash step was performed after adapter ligation. The samples were sequenced on an Illumina MiSeq to generate 2 × 300-bp sequence reads.

### Data analysis for Illumina sequencing.

Raw sequence reads were quality controlled using fastp (48). The quality-controlled reads were normalized using bbnorm (49) and subsequently *de novo* assembled using SPAdes (50). Minimap2 (51) was used to align the quality-controlled reads against the obtained contigs and the obtained bam files were loaded into Geneious (52) for minor variant determination with a 20% cutoff.

### Sequencing alignment.

Each segment of viral genome was aligned using the CLUSTAL W algorithm in MEGA5 (53). Next, sequences of all individual isolated were aligned using the BioEdit version 7.0. The 5′ untranslated region or noncoding region was resected from sequences.

### Virus attachment.

Cell suspensions of SK-N-SH or RD cells were incubated with EV-D68 strains for 1 h at 4°C using an MOI of 1. As a negative control, viruses were heat inactivated at 62°C for 10 min before incubation with the cells. Subsequently, cells were washed with PBS, fixed with 4% PFA for 15 min, and blocked for 30 min with PBS containing 5% normal goat serum (Dako, Denmark). Cells were incubated with rabbit anti-EV-D68 VP1 (20 μg/ml; GeneTex) in 2 mM EDTA (Sigma-Aldrich) and 0.1% BSA (Aurion) fluorescence-activated cell sorting (FACS) buffer for 1 h at 4°C. After washing 3 times, cells were incubated with a secondary goat-anti rabbit IgG conjugated to Alexa Fluor 594 (10 μg/ml; Life Technologies) in FACS buffer. After incubation, cells were washed 3 times in FACS buffer and analyzed using a BD FACS Lyrics flow cytometer (BD Bioscience, USA). The percentage of cells to which virus attached was determined using FlowJo 10 software (Ashland, OR, USA). Experiments were performed at least 3 times, and each experiment was performed in duplicate.

### Removal of cell surface sialic acid and heparan sulfate.

Cell suspensions of SK-N-SH or RD cells were incubated with 100 mU/ml Arthrobacter ureafaciens neuraminidase (Roche) or 10 mIU/ml heparinase III (Sigma-Aldrich) in serum-free medium for 1 h at 37°C. To prevent the cell surface sulfation, cells were cultured for 5 days with 80 mM sodium chlorate (NaClO_3_; Sigma-Aldrich, 1.06420 EMD Millipore). Removal of α(2,3)-linked sialic acid and α(2,6)-linked sialic acid on the cell surface was confirmed by staining with fluorescein-labeled Sambucus nigra lectin (SNA) (5 μg/ml; Vector Laboratories, CA, USA) and biotinylated Maackia amurensis lectin (MAL) I (5 μg/ml; Vector Laboratories). The sialylated cells were detected by streptavidin-conjugated Alexa Fluor 488 (5 μg/ml; Thermo Fisher Scientific). Removal of heparan sulfate on the cell surface was confirmed using a mouse anti-heparan sulfate monoclonal antibody (10 μg/ml; Amsbio, Frankfurt, Germany). No-virus inoculation control and virus inoculation in nonenzymatic treatment control were included as a negative control and positive control, respectively, in all assays. Mean fluorescence intensity (MFI) was measured with BD FACS Lyrics (BD Bioscience). Subsequently, virus attachment was determined as described above. Data were analyzed using FlowJo 10 software (Ashland, OR, USA). The experiments were performed at least 3 times, and each experiment was performed in duplicate.

### RT-qPCR.

Total nucleic acid was extracted from cell lysis using the Magna Pure MagNA Pure LC total nucleic acid isolation kit (Roche) according to manufacturer’s instructions. The total nucleic acid was eluted in 50 μl. For viral RNA quantification, a real-time TaqMan RT-PCR assay was performed using the Applied Biosystems 7500 real-time PCR system (Thermo Fisher Scientific). The experiments were carried out by adding forward and reverse EV-D68 primer (75 pmol/μl) and the probes (10 pmol/μl). The following sequences including EV-D68 specific primer and probe were used: forward primer 5′-TGTTCCCACGGTTGAAAACAA-3′, reverse primer 5′-TGTCTAGCGTCTCATGGTTTTCAC-3′, probe 1 5′-TCCGCTATAGTACTTCG-3′, and probe 2 5′-ACCGCTATAGTACTTCG-3′. The following reaction conditions were applied for all PCR experiments: 5 min at 50°C and 20 s at 95°C, followed by 45 cycles at 95°C for 3 s, and 60°C for 31 s.

### Statistical analyses.

Statistical analyses were performed using GraphPad Prism 6.0 software (La Jolla, CA, USA). Specific tests are described in the figure legends*. P* values of ≤0.05 were considered significant. All data were expressed as means ± standard deviations (SDs). Experiments were performed at least in biological triplicates and technical duplicates.

### Data availability.

The viruses included in this study are available under accession numbers MN954536, MN954537, MN954538, MN954539, MN954540, and MN954541.

## References

[1] Imamura T, Oshitani H 2015 Global reemergence of enterovirus D68 as an important pathogen for acute respiratory infections. Rev Med Virol 25:102–114. doi:10.1002/rmv.1820. 25471236 PMC4407910

[2] Messacar K, Schreiner TL, Maloney JA, Wallace A, Ludke J, Oberste MS, Nix WA, Robinson CC, Glodé MP, Abzug MJ, Dominguez SR 2015 A cluster of acute flaccid paralysis and cranial nerve dysfunction temporally associated with an outbreak of enterovirus D68 in children in Colorado, USA. Lancet 385:1662–1671. doi:10.1016/S0140-6736(14)62457-0. 25638662

[3] Greninger AL, Naccache SN, Messacar K, Clayton A, Yu G, Somasekar S, Federman S, Stryke D, Anderson C, Yagi S, Messenger S, Wadford D, Xia D, Watt JP, Van Haren K, Dominguez SR, Glaser C, Aldrovandi PG, Chiu DCY 2015 A novel outbreak enterovirus D68 strain associated with acute flaccid myelitis cases in the USA (2012–14): a retrospective cohort study. Lancet Infect Dis 15:671–682. doi:10.1016/S1473-3099(15)70093-9. 25837569 PMC6027625

[4] Holm-Hansen CC, Midgley SE, Fischer TK 2016 Global emergence of enterovirus D68: a systematic review. Lancet Infect Dis 16:e64–e75. doi:10.1016/S1473-3099(15)00543-5. 26929196

[5] Dyda A, Stelzer-Braid S, Adam D, Chughtai AA, MacIntyre CR 2018 The association between acute flaccid myelitis (AFM) and Enterovirus D68 (EV-D68) - what is the evidence for causation? Euro Surveill 23:17-00310. doi:10.2807/1560-7917.ES.2018.23.3.17-00310. PMC5792700 29386095

[6] Knoester M, Scholvinck EH, Poelman R, Smit S, Vermont CL, Niesters HG, Van Leer-Buter CC 2017 Upsurge of Enterovirus D68, the Netherlands, 2016. Emerg Infect Dis 23:140–143. doi:10.3201/eid2301.161313. 27660916 PMC5176244

[7] Furuse Y, Chaimongkol N, Okamoto M, Imamura T, Saito M, Tamaki R, Saito M, Tohoku-RITM Collaborative Research Team, Lupisan SP, Oshitani H 2015 Molecular epidemiology of enterovirus D68 from 2013 to 2014 in Philippines. J Clin Microbiol 53:1015–1018. doi:10.1128/JCM.03362-14. 25568441 PMC4390660

[8] Messacar K, Abzug MJ, Dominguez SR 2016 2014 outbreak of enterovirus D68 in North America. J Med Virol 88:739–745. doi:10.1002/jmv.24410. 26489019

[9] Midgley CM, Jackson MA, Selvarangan R, Turabelidze G, Obringer E, Johnson D, Giles BL, Patel A, Echols F, Oberste MS, Nix WA, Watson JT, Gerber SI 2014 Severe respiratory illness associated with enterovirus D68 - Missouri and Illinois, 2014. MMWR Morb Mortal Wkly Rep 63:798–799. 25211545 PMC4584696

[10] Zhang Y, Cao J, Zhang S, Lee AJ, Sun G, Larsen CN, Zhao H, Gu Z, He S, Klem EB, Scheuermann RH 2016 Genetic changes found in a distinct clade of Enterovirus D68 associated with paralysis during the 2014 outbreak. Virus Evol 2:vew015. doi:10.1093/ve/vew015. 28512577 PMC5426007

[11] Yogo N, Imamura T, Muto Y, Hirai K 2019 Cardiopulmonary failure as a result of brainstem encephalitis caused by enterovirus D68. BMJ Case Rep 12:e231990. doi:10.1136/bcr-2019-231990. PMC6887471 31732545

[12] Esposito S, Chidini G, Cinnante C, Napolitano L, Giannini A, Terranova L, Niesters H, Principi N, Calderini E 2017 Acute flaccid myelitis associated with enterovirus-D68 infection in an otherwise healthy child. Virol J 14:4. doi:10.1186/s12985-016-0678-0. 28081720 PMC5234096

[13] Kreuter JD, Barnes A, McCarthy JE, Schwartzman JD, Oberste MS, Rhodes CH, Modlin JF, Wright PF 2011 A fatal central nervous system enterovirus 68 infection. Arch Pathol Lab Med 135:793–796. doi:10.1043/2010-0174-CR.1. 21631275

[14] Messacar K, Pretty K, Reno S, Dominguez SR 2019 Continued biennial circulation of enterovirus D68 in Colorado. J Clin Virol 113:24–26. doi:10.1016/j.jcv.2019.01.008. 30825833

[15] Carballo CM, Erro MG, Sordelli N, Vazquez G, Mistchenko AS, Cejas C, Rodriguez M, Cisterna DM, Freire MC, Contrini MM, Lopez EL 2019 Acute flaccid myelitis associated with enterovirus D68 in children, Argentina, 2016. Emerg Infect Dis 25:573–576. doi:10.3201/eid2503.170897. 30602120 PMC6390768

[16] Pellegrinelli L, Giardina F, Lunghi G, Uceda Renteria SC, Greco L, Fratini A, Galli C, Piralla A, Binda S, Pariani E, Baldanti F 2019 Emergence of divergent enterovirus (EV) D68 sub-clade D1 strains, northern Italy, September to October 2018. Euro Surveill 24:1900090. doi:10.2807/1560-7917.ES.2018.24.7.1900090. PMC6381661 30782269

[17] The United Kingdom Acute Flaccid Paralysis Afp Task Force. 2019 An increase in reports of acute flaccid paralysis (AFP) in the United Kingdom, 1 January 2018–21 January 2019: early findings. Euro Surveill 24:1900093. doi:10.2807/1560-7917.ES.2019.24.6.1900093. PMC6373064 30755296

[18] Cathcart AL, Baggs EL, Semler BL 2015 Picornaviruses: pathogenesis and molecular biology. Elsevier Inc, University of California, Irvine, CA, USA.

[19] Baggen J, Thibaut HJ, Staring J, Jae LT, Liu Y, Guo H, Slager JJ, de Bruin JW, van Vliet ALW, Blomen VA, Overduin P, Sheng J, de Haan CAM, de Vries E, Meijer A, Rossmann MG, Brummelkamp TR, van Kuppeveld FJM 2016 Enterovirus D68 receptor requirements unveiled by haploid genetics. Proc Natl Acad Sci U S A 113:1399–1404. doi:10.1073/pnas.1524498113. 26787879 PMC4747778

[20] Wei W, Guo H, Chang J, Yu Y, Liu G, Zhang N, Willard SH, Zheng S, Yu XF 2016 ICAM-5/telencephalin is a functional entry receptor for enterovirus D68. Cell Host Microbe 20:631–641. doi:10.1016/j.chom.2016.09.013. 27923705

[21] Baggen J, Liu Y, Lyoo H, van Vliet ALW, Wahedi M, de Bruin JW, Roberts RW, Overduin P, Meijer A, Rossmann MG, Thibaut HJ, van Kuppeveld FJM 2019 Bypassing pan-enterovirus host factor PLA2G16. Nat Commun 10:3171. doi:10.1038/s41467-019-11256-z. 31320648 PMC6639302

[22] Wei HY, Yeh TK, Hsieh JY, Lin IP, Yang JY 2018 Updates on the molecular epidemiology of Enterovirus D68 after installation of screening test among acute flaccid paralysis patients in Taiwan. J Microbiol Immunol Infect 51:688–691. doi:10.1016/j.jmii.2017.12.001. 29339008

[23] Fall A, Jallow MM, Kebe O, Kiori DE, Sy S, Goudiaby D, Boye CSB, Niang MN, Dia N 2019 Low circulation of subclade A1 enterovirus D68 strains in Senegal during 2014 North America outbreak. Emerg Infect Dis 25:1404–1407. doi:10.3201/eid2507.181441. 31211670 PMC6590772

[24] King AMQ, Adams MJ, Carstens EB, Lefkowitz EJ (ed). 2012 Virus taxonomy: ninth report of the International Committee on Taxonomy of Viruses. Elsevier, New York, NY.

[25] Yip CCY, Lo JYC, Sridhar S, Lung DC, Luk S, Chan KH, Chan JFW, Cheng VCC, Woo PCY, Yuen KY, Lau SKP 2017 First report of a fatal case associated with EV-D68 infection in Hong Kong and emergence of an interclade recombinant in China revealed by genome analysis. Int J Mol Sci 18:1065. doi:10.3390/ijms18051065. PMC5454976 28509856

[26] Messacar K, Schreiner TL, Van Haren K, Yang M, Glaser CA, Tyler KL, Dominguez SR 2016 Acute flaccid myelitis: a clinical review of US cases 2012–2015. Ann Neurol 80:326–338. doi:10.1002/ana.24730. 27422805 PMC5098271

[27] Wang G, Zhuge J, Huang W, Nolan SM, Gilrane VL, Yin C, Dimitrova N, Fallon JT 2017 Enterovirus D68 subclade B3 strain circulating and causing an outbreak in the United States in 2016. Sci Rep 7:1242. doi:10.1038/s41598-017-01349-4. 28455514 PMC5430842

[28] Dyrdak R, Grabbe M, Hammas B, Ekwall J, Hansson KE, Luthander J, Naucler P, Reinius H, Rotzen-Ostlund M, Albert J 2016 Outbreak of enterovirus D68 of the new B3 lineage in Stockholm, Sweden, August to September 2016. Euro Surveill 21:30403. doi:10.2807/1560-7917.ES.2016.21.46.30403. PMC5144949 27918255

[29] Piralla A, Principi N, Ruggiero L, Girello A, Giardina F, De Sando E, Caimmi S, Bianchini S, Marseglia GL, Lunghi G, Baldanti F, Esposito S 2018 Enterovirus-D68 (EV-D68) in pediatric patients with respiratory infection: the circulation of a new B3 clade in Italy. J Clin Virol 99–100:91–96. doi:10.1016/j.jcv.2018.01.005. PMC7185653 29396353

[30] Rosenfeld AB, Warren AL, Racaniello VR 2019 Neurotropism of enterovirus D68 isolates is independent of sialic acid and is not a recently acquired phenotype. mBio 10:e02370-19. doi:10.1128/mBio.02370-19. 31641090 PMC6805996

[31] Hixon AM, Yu G, Leser JS, Yagi S, Clarke P, Chiu CY, Tyler KL 2017 A mouse model of paralytic myelitis caused by enterovirus D68. PLoS Pathog 13:e1006199. doi:10.1371/journal.ppat.1006199. 28231269 PMC5322875

[32] Tan CW, Poh CL, Sam IC, Chan YF 2013 Enterovirus 71 uses cell surface heparan sulfate glycosaminoglycan as an attachment receptor. J Virol 87:611–620. doi:10.1128/JVI.02226-12. 23097443 PMC3536405

[33] Hixon AM, Clarke P, Tyler KL 2019 Contemporary circulating enterovirus D68 strains infect and undergo retrograde axonal transport in spinal motor neurons independent of sialic acid. J Virol 93:e00578-19. doi:10.1128/JVI.00578-19. 31167912 PMC6675884

[34] Bochkov YA, Watters K, Basnet S, Sijapati S, Hill M, Palmenberg AC, Gern JE 2016 Mutations in VP1 and 3A proteins improve binding and replication of rhinovirus C15 in HeLa-E8 cells. Virology 499:350–360. doi:10.1016/j.virol.2016.09.025. 27743961 PMC5110265

[35] Goodfellow IG, Sioofy AB, Powell RM, Evans DJ 2001 Echoviruses bind heparan sulfate at the cell surface. J Virol 75:4918–4921. doi:10.1128/JVI.75.10.4918-4921.2001. 11312365 PMC114248

[36] Tan CW, Sam IC, Lee VS, Wong HV, Chan YF 2017 VP1 residues around the five-fold axis of enterovirus A71 mediate heparan sulfate interaction. Virology 501:79–87. doi:10.1016/j.virol.2016.11.009. 27875780

[37] Brown DM, Hixon AM, Oldfield LM, Zhang Y, Novotny M, Wang W, Das SR, Shabman RS, Tyler KL, Scheuermann RH 2018 Contemporary circulating enterovirus D68 strains have acquired the capacity for viral entry and replication in human neuronal cells. mBio 9:e01954-18. doi:10.1128/mBio.01954-18. 30327438 PMC6191546

[38] Tseligka ED, Sobo K, Stoppini L, Cagno V, Abdul F, Piuz I, Meylan P, Huang S, Constant S, Tapparel C 2018 A VP1 mutation acquired during an enterovirus 71 disseminated infection confers heparan sulfate binding ability and modulates ex vivo tropism. PLoS Pathog 14:e1007190. doi:10.1371/journal.ppat.1007190. 30075025 PMC6093697

[39] Jenniskens GJ, Oosterhof A, Brandwijk R, Veerkamp JH, van Kuppevelt TH 2000 Heparan sulfate heterogeneity in skeletal muscle basal lamina: demonstration by phage display-derived antibodies. J Neurosci 20:4099–4111. doi:10.1523/JNEUROSCI.20-11-04099.2000. 10818145 PMC6772625

[40] Karelehto E, Koen G, Benschop K, van der Klis F, Pajkrt D, Wolthers K 2019 Enterovirus D68 serosurvey: evidence for endemic circulation in the Netherlands, 2006 to 2016. Euro Surveill 24:1800671. doi:10.2807/1560-7917.ES.2019.24.35.1800671. PMC6724466 31481149

[41] Harrison CJ, Weldon WC, Pahud BA, Jackson MA, Oberste MS, Selvarangan R 2019 Neutralizing antibody against enterovirus D68 in children and adults before 2014 outbreak, Kansas City, Missouri, USA. Emerg Infect Dis 25:585–588. doi:10.3201/eid2503.180960. 30789123 PMC6390745

[42] Schubert RD, Hawes IA, Ramachandran PS, Ramesh A, Crawford ED, Pak JE, Wu W, Cheung CK, O’Donovan BD, Tato CM, Lyden A, Tan M, Sit R, Sowa GA, Sample HA, Zorn KC, Banerji D, Khan LM, Bove R, Hauser SL, Gelfand AA, Johnson-Kerner BL, Nash K, Krishnamoorthy KS, Chitnis T, Ding JZ, McMillan HJ, Chiu CY, Briggs B, Glaser CA, Yen C, Chu V, Wadford DA, Dominguez SR, Ng TFF, Marine RL, Lopez AS, Nix WA, Soldatos A, Gorman MP, Benson L, Messacar K, Konopka-Anstadt JL, Oberste MS, DeRisi JL, Wilson MR 2019 Pan-viral serology implicates enteroviruses in acute flaccid myelitis. Nat Med 25:1748–1752. doi:10.1038/s41591-019-0613-1. 31636453 PMC6858576

[43] Mishra N, Ng TFF, Marine RL, Jain K, Ng J, Thakkar R, Caciula A, Price A, Garcia JA, Burns JC, Thakur KT, Hetzler KL, Routh JA, Konopka-Anstadt JL, Nix WA, Tokarz R, Briese T, Oberste MS, Lipkin WI 2019 Antibodies to enteroviruses in cerebrospinal fluid of patients with acute flaccid myelitis. mBio 10:e01903-19. doi:10.1128/mBio.01903-19. 31409689 PMC6692520

[44] Okumura A, Numoto S, Iwayama H, Kurahashi H, Natsume J, Saitoh S, Yoshikawa T, Fukao T, Hirayama M, Takahashi Y 2020 Respiratory illness and acute flaccid myelitis in the Tokai district in 2018. Pediatr Int 62:337–340. doi:10.1111/ped.14128. 31886594

[45] Meijer A, Benschop KS, Donker GA, van der Avoort HG 2014 Continued seasonal circulation of enterovirus D68 in the Netherlands, 2011–2014. Euro Surveill 19:20935. doi:10.2807/1560-7917.es2014.19.42.20935. 25358039

[46] Meijer A, van der Sanden S, Snijders BE, Jaramillo-Gutierrez G, Bont L, van der Ent CK, Overduin P, Jenny SL, Jusic E, van der Avoort HG, Smith GJ, Donker GA, Koopmans MP 2012 Emergence and epidemic occurrence of enterovirus 68 respiratory infections in The Netherlands in 2010. Virology 423:49–57. doi:10.1016/j.virol.2011.11.021. 22177700

[47] Kärber G 1931 Beitrag zur kollektiven Behandlung pharmakologischer Reihenversuche. Naunyn Schmiedebergs Arch Exp Pathol Pharmakol 162:480–483. doi:10.1007/BF01863914.

[48] Nikolay B, Weidmann M, Dupressoir A, Faye O, Boye CS, Diallo M, Sall AA 2014 Development of a Usutu virus specific real-time reverse transcription PCR assay based on sequenced strains from Africa and Europe. J Virol Methods 197:51–54. doi:10.1016/j.jviromet.2013.08.039. 24036076

[49] Bushnell B 2018 BBMap download. https://sourceforge.net/projects/bbmap/. Accessed 9 August 2019.

[50] Bankevich A, Nurk S, Antipov D, Gurevich AA, Dvorkin M, Kulikov AS, Lesin VM, Nikolenko SI, Pham S, Prjibelski AD, Pyshkin AV, Sirotkin AV, Vyahhi N, Tesler G, Alekseyev MA, Pevzner PA 2012 SPAdes: a new genome assembly algorithm and its applications to single-cell sequencing. J Comput Biol 19:455–477. doi:10.1089/cmb.2012.0021. 22506599 PMC3342519

[51] Li H 2018 Minimap2: pairwise alignment for nucleotide sequences. Bioinformatics 34:3094–3100. doi:10.1093/bioinformatics/bty191. 29750242 PMC6137996

[52] Kearse M, Moir R, Wilson A, Stones-Havas S, Cheung M, Sturrock S, Buxton S, Cooper A, Markowitz S, Duran C, Thierer T, Ashton B, Meintjes P, Drummond A 2012 Geneious Basic: an integrated and extendable desktop software platform for the organization and analysis of sequence data. Bioinformatics 28:1647–1649. doi:10.1093/bioinformatics/bts199. 22543367 PMC3371832

[53] Tamura K, Peterson D, Peterson N, Stecher G, Nei M, Kumar S 2011 MEGA5: molecular evolutionary genetics analysis using maximum likelihood, evolutionary distance, and maximum parsimony methods. Mol Biol Evol 28:2731–2739. doi:10.1093/molbev/msr121. 21546353 PMC3203626

