# Erratum for Sooksawasdi Na Ayudhya et al., “Enhanced Enterovirus D68 Replication in Neuroblastoma Cells Is Associated with a Cell Culture-Adaptive Amino Acid Substitution in VP1”

**DOI:** 10.1128/mSphere.01147-20

**Published:** 2020-12-09

**Authors:** Syriam Sooksawasdi Na Ayudhya, Adam Meijer, Lisa Bauer, Bas Oude Munnink, Carmen Embregts, Lonneke Leijten, Jurre Y. Siegers, Brigitta M. Laksono, Frank van Kuppeveld, Thijs Kuiken, Corine GeurtsvanKessel, Debby van Riel

**Affiliations:** a Department of Viroscience, Erasmus MC, Rotterdam, The Netherlands; b National Institute for Public Health and the Environment, Centre for Infectious Diseases Research, Diagnostics and Laboratory Surveillance, Bilthoven, The Netherlands; c Virology Division, Department of Infectious Diseases and Immunology, Faculty of Veterinary Medicine, Utrecht University, Utrecht, The Netherlands

## ERRATUM

Volume 5, no. 6, e00941-20, 2020, https://doi.org/10.1128/mSphere.00941-20. Page 1: Corine GeurtsvanKessel’s surname was misspelled as “Geurts-van Kessel” in the byline and was corrected on 6 November 2020.

