# Supplementary figures and images for: Enhanced Enterovirus D68 Replication in Neuroblastoma Cells Is Associated with a Cell Culture-Adaptive Amino Acid Substitution in VP1
Source: mSphere. 2020 Nov 4;5(6):e00941-20. doi: 10.1128/mSphere.00941-20 (PMC7643833; doi:10.1128/mSphere.00941-20)

**Fig S.1.**


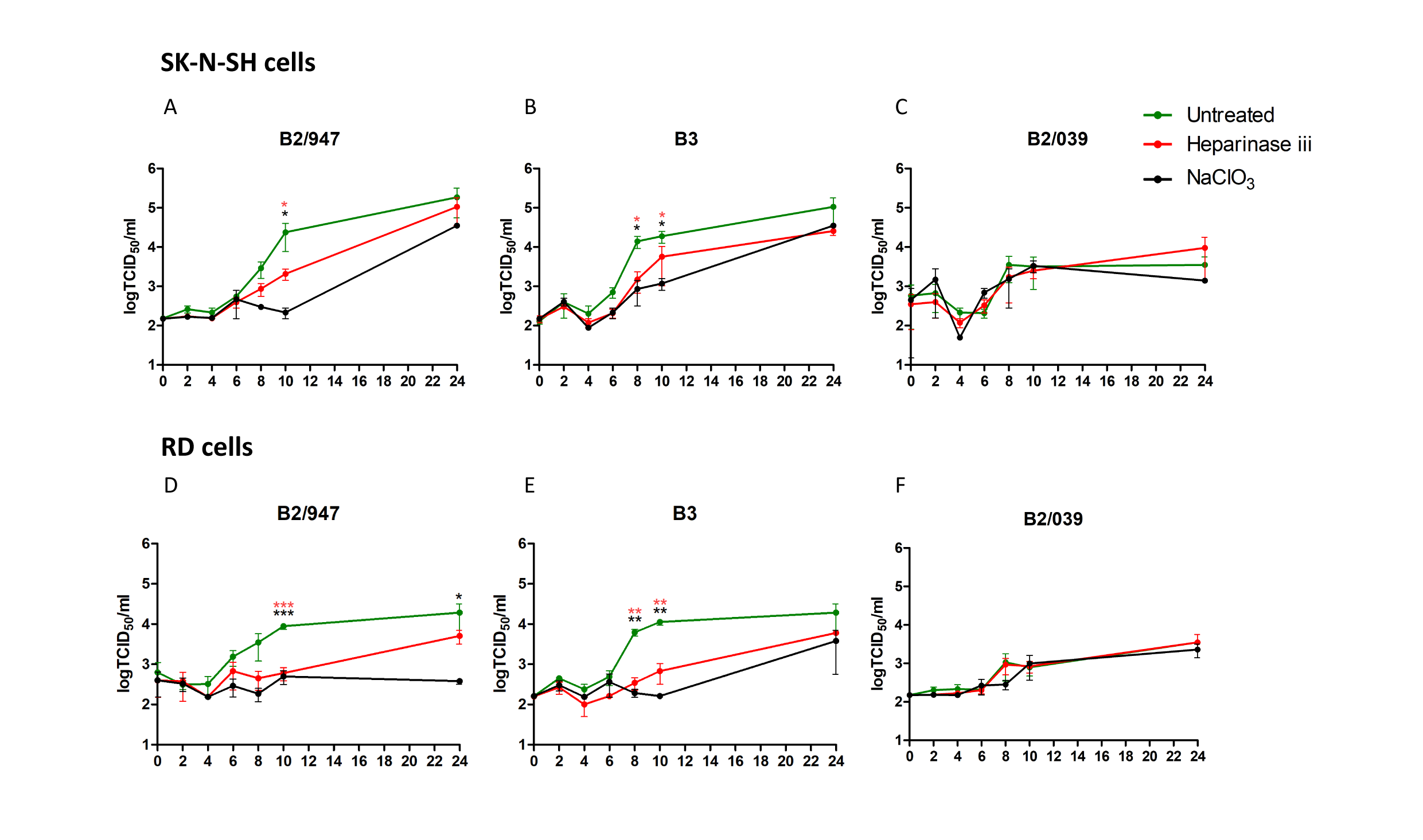

Supplement: FIG S1 [file mSphere.00941-20-sf001.docx]
